# Comparative effectiveness of telehealth-delivered family and standard models of diabetes self-management education and support for persons with type 2 diabetes mellitus^☆^

**DOI:** 10.1016/j.diabres.2025.113063

**Published:** 2025-12-20

**Authors:** Jennifer A. Andersen, Pearl A. McElfish, James P. Selig, Ji Li, Brett Rowland, Jonell Hudson, Shashank Kraleti, Joseph Henske, Lindsay S. Mayberry

**Affiliations:** a College of Medicine, University of Arkansas for Medical Sciences Northwest, 2708 S. 48th St., Springdale, AR 72762, USA; b Institute for Community Health Innovation, University of Arkansas for Medical Sciences Northwest, 2708 S. 48th St., Springdale, AR 72762, USA; c Fay W. Boozman College of Public Health, University of Arkansas for Medical Sciences Northwest, 2708 S. 48th St., Springdale, AR 72762, USA; d College of Pharmacy, University of Arkansas for Medical Sciences Northwest, 1125 N. College Ave., Fayetteville, AR 72703, USA; e College of Medicine, University of Arkansas for Medical Sciences, 4301 W. Marksham St., Little Rock, AR 72205, USA; f Department of Medicine, Vanderbilt University Medical Center, 1211 Medical Center Dr., Nashville, TN 37232, USA

**Keywords:** Type 2 diabetes mellitus, Diabetes self-management, Family model, HbA1c, Telehealth

## Abstract

**Aims:**

Family members/support persons can shape the self-management decisions of persons with T2DM (PWDs), and their inclusion in DSMES may lead to improved outcomes. However, research on the effectiveness of family models of DSMES (Family-DSMES) is limited. We conducted a comparative effectiveness-implementation RCT of Family-DSMES and a standard model of DSMES (Standard-DSMES) delivered via telehealth.

**Methods:**

550 diverse adult PWDs and their family members/support persons were recruited from eight primary care clinics in both rural and urban areas. Process data was collected to evaluate implementation characteristics. Biometric, behavioral, and psychosocial measures were collected from PWDs.

**Results:**

Mean attendance was slightly higher for PWDs in Family-DSMES than Standard-DSMES (62 % vs. 57 %, *p* = 0.096). Engagement (*p* = 0.319) and class time (*p* = 0.145) were similar across arms. PWDs in both arms experienced a decrease in HbA1c at all time points (*p* < 0.005). No statistically significant between-arm differences were found for biometric outcomes or behavioral outcomes including HbA1c. For diabetes-related helpful family involvement, the improvement in the Family-DSMES arm was significantly greater than in the Standard-DSMES arm (*p* = 0.024).

**Conclusions:**

Results demonstrate the ability to engage rural and diverse PWDs in a telehealth intervention with high fidelity to achieve clinically and statistically significant improvements in HbA1c.

## 1. Introduction

Type 2 diabetes mellitus (T2DM) affects approximately 11 % of individuals in the United States (US) [1], including an estimated 15 % (363,781 people) of Arkansas’ population [2]. Persons with type 2 diabetes mellitus (PWDs) who consistently engage in daily self-management practices are more likely to have better glycemic management, improve cardiovascular health, and reduce the risk of diabetes-related complications; [3,4] however, in the US, only half of PWDs meet glycemic goals [5].

A recent consensus report [4] published by seven professional organizations advocated for diabetes self-management education and support (DSMES) to assist PWDs in adopting essential self-management practices and enhancing health outcomes [4,6]. DSMES provides guidance on several aspects of diabetes care, including healthy eating, being active, glucose monitoring, understanding blood glucose, taking medications, problem solving, reducing risk, healthy coping, mitigating complications of diabetes, and goal setting [7]. A ten-hour DSMES program is recognized as part of the standard of care for PWDs [6,8]. DSMES has demonstrated effectiveness in improving key diabetes-related clinical measures, including hemoglobin A1c (HbA1c), body mass index (BMI), blood pressure, and lipids [9,10]. DSMES is also effective at improving diabetes self-efficacy and empowerment [11], diabetes-related quality of life [12,13], healthy coping skills [14], and diabetes self-management behaviors [15] and at reducing diabetes-related complications [16], diabetes-related distress [17,18], and depression [19]. These improvements extend to DSMES delivered by telehealth as well, which has been shown to reduce PWDs’ HbA1c and to improve their diabetes knowledge [20–22].

Despite the demonstrated benefits of DSMES, many PWDs either do not attend DSMES or attend a limited number of classes [8]. Family members/support persons can shape the self-management decisions of PWDs through their communication, daily habits, and overall attitudes toward diabetes. The lack of DSMES attendance and the inability to meet glycemic goals may stem from a misalignment between individual education approaches and the lived experiences of PWDs who navigate self-management within their interpersonal and social environments [23–29]. Family members/support persons may also pose challenges to effective diabetes self-management through their food shopping and preparing, activity patterns, communication styles, and competing priorities [23–29]. Beneficial and detrimental aspects of family involvement often coexist [25,30–32], suggesting that without appropriate psychoeducation on providing support, family members/support persons may inadvertently undermine self-care efforts [25,30,33] or engage in conflicts over diabetes management [25,31]. Therefore, incorporating family members/support persons into DSMES and engaging them in diabetes management in helpful and autonomy-supportive ways may lead to greater improvements in outcomes [23–28,34,35].

Systematic reviews have identified over 20 studies demonstrating the effectiveness of family-centered DSMES models [27,28,36] on outcomes including glycemic management, self-care behaviors, support for diabetes, PWDs and family quality of life, and diabetes distress. However, gaps in the literature remain [37]. Most prior studies have focused on one racial/ethnic group and have used culturally adapted curricula. Furthermore, the duration of Family-DSMES curricula evaluated varies significantly, and many do not align with the guidelines established by the Centers for Medicare and Medicaid Services, the American Diabetes Association, and the Association of Diabetes Care & Education Specialists [4], which hinders their widespread adoption and replicability. No existing research, to our knowledge, has examined if Family-DSMES can be implemented without increasing delivery burden relative to Standard-DSMES, while successfully meeting standards set forth for DSMES program accreditation [38] and adhering to the ten-hour limit covered by Medicare, Medicaid, and most insurance [8]. Furthermore, prior studies have not delivered Family-DSMES via telehealth, constraining knowledge on viable models for scalability.

We sought to address these gaps in the literature by conducting a fully powered randomized controlled trial (RCT) comparing Family-DSMES to Standard-DSMES using a ten-hour DSMES curricula that was delivered via telehealth. First, we examined process data to determine if implementation characteristics (attendance, engagement, fidelity) of Family-DSMES were like Standard-DSMES, and if Family-DMSES was achievable within the ten hours covered by insurance while meeting established national standards. Second, we examined the effects of Standard- and Family-DSMES on PWDs’ biometric outcomes—including our primary outcome of change in HbA1c from preintervention to immediate post-intervention—and behavioral and psychosocial outcomes. Third, across Standard- and Family-DSMES, we compared effects on outcomes and explored the heterogeneous treatment effects on HbA1c and diabetes-related emotional distress at immediate post-intervention.

## 2. Materials and methods

This study was a two-arm comparative effectiveness-implementation RCT of two diabetes education interventions delivered via telehealth: 1) Family-DSMES and 2) Standard-DSMES and their effect on diabetes outcomes. The RCT protocol, including full descriptions of both study arms, has been published [39]. All study procedures were approved by the University of Arkansas for Medical Sciences Institutional Review Board (#260059), and the study is registered with ClinicalTrials.gov (#NCT04334109).

### 2.1. Study participants

Between 2021 and 2022, we recruited 550 adult PWDs from 8 primary care clinics across Arkansas affiliated with the Rural Research Network [40]. These clinics serve a diverse patient population with more Black and Hispanic patients than the general population of Arkansas. Clinic locations were diverse as well, with half of the participating clinics in rural areas and half in more urban locations.

Eligibility included 1) age eighteen years or older; 2) diagnosis of T2DM with current HbA1c ≥ 7.0 %; 3) ability to speak/read English; and 4) having a family member/support person assisting the PWD with household matters willing to participate in the study. Exclusion criteria included 1) having received formal DSMES in the past three years; 2) having a condition which precludes full participation, such as a terminal illness; and 3) having plans to move out of the area during the study.

Initial eligibility was determined through medical record abstraction. Patients who were deemed potentially eligible were sent a recruitment letter from their clinic and the study principal investigator with information about the study, followed by a phone call from study staff to discuss interest in the study. For this study, a family member/support person was defined as “a person living in the same household and/or assisting the person with diabetes with household matters.” Family members/support persons were eligible if they were eighteen or older and able to speak/read in English. Family members/support persons in both arms were invited to participate in the study and were included in all data collection activities. See protocol paper for additional details [39].

### 2.2. Study design and procedure

The enrollment visit took place in a private room at the PWD’s home clinic. Both the PWD and family member/support person provided written consent. Baseline data collection was completed immediately after enrollment for both PWDs and family members/support persons in both arms. PWDs and family members/support persons were each compensated with a $100 Walmart gift card for each of the three data collection study visits (baseline, immediate post-intervention, 6 months post-intervention; up to $300 per person). All PWDs signed a medical records release for data abstraction at 12 months post-intervention.

#### 2.2.1. Randomization

Dyads, consisting of a PWD and family member/support person, were randomized at a ratio of 1:1 to either Family-DSMES or Standard-DSMES. Randomization was based on a stratified permuted block approach with block sizes of 2, 4, and 6. Strata included PWD gender, age, and race/ethnicity. Allocation lists were generated by the study biostatistician using the R package blockrand and then uploaded to REDCap. Assignment was concealed until after enrollment procedures and baseline data collection were completed

DSMES classes were scheduled based on availability of the PWD. PWDs randomized to the Family-DSMES were asked to attend sessions with the family member/support person enrolled in the study, and PWDs in the Standard-DSMES were asked to attend sessions alone. In both arms, DSMES sessions occurred once weekly for ten weeks and were delivered by a certified diabetes care and education specialist (CDCES).

#### 2.2.2. Data collection

Biometric measures collected at baseline and immediate post-intervention included HbA1c, non-fasting blood glucose and lipids, weight and height to calculate BMI, and blood pressure for both PWDs and family members/support persons in both arms. HbA1c, BMI, and blood pressure were measured again at the 6 months post-intervention study visit for both PWD and family member/support person participants. PWD HbA1c was collected through medical record abstraction at 12 months post-intervention. PWDs completed surveys at study visits at baseline and immediate post-intervention, which included measures of sociodemographic characteristics, diabetes self-care behaviors, glucose-lowering medication adherence, diabetes self-efficacy, diabetes-related emotional distress, diabetes-related helpful and harmful family behaviors, and diabetes-related quality of life. Family members/support persons completed a slightly shorter survey at study visits at baseline and immediate post-intervention. Effects on family members/support persons will be described in a separate paper

### 2.3. Study arms

Both Family-DSMES and Standard-DSMES used the same evidence-based curriculum, “Type 2 Diabetes Basics,” published by the International Diabetes Center, which meets standards for DSMES accreditation and adheres to the ten hours of DSMES covered by Medicare, Medicaid, and most insurances. DSMES topics were consistent with National Standards and included healthy eating, being active, glucose monitoring, taking medication, problem solving, reducing risks, healthy coping, and goal setting. Differences in Family-DSMES curriculum were: 1) family members/support persons were encouraged to attend DSMES sessions, 2) didactic curriculum provided psychoeducation on how to support the PWD in managing their diabetes, and 3) goal setting sessions had an additional family goal setting activity. The protocol publication has additional details on Family- and Standard-DSMES curricula [39].

Family-DSMES and Standard-DSMES sessions were conducted in a small-group format via telehealth using Zoom for Healthcare in ten weekly one-hour group sessions to total approximately ten hours. Each group consisted of four to six PWDs, the CDCES, and a class assistant who assisted with technical issues as needed—plus family members/support persons of PWDs in Family-DSMES. All PWDs received an internet-capable iPad for the duration of the intervention period to ensure they could participate in DSMES sessions. PWDs without access to the internet were provided with a cellular data plan. Family members/support persons assigned to Family-DSMES who did not live with the PWD also received an iPad and cellular data support if needed to support their attendance. A total of 152 PWDs and 14 family members/support persons were provided with cellular data plans.

#### 2.3.1. Goal setting

CDCESs underwent six hours of training on family goal setting. Goal setting occurred in sessions three, seven, and ten in both study arms. PWDs in both study arms worked with the CDCESs to set a behavioral SMART (specific, measurable, actionable, realistic, and time-bound) goal in the first goal setting session. CDCESs were trained to work with the PWD to make sure the goal was behavioral (rather than an outcome) and that the PWD felt confident they could achieve their goal four or more days per week to ensure the goal was realistic. Progress on this goal was reviewed in subsequent goal setting sessions, and PWDs had the option to add to or change the SMART goal

During goal setting for Family-DSMES, PWDs and their family members/support persons were placed together in a Zoom breakout room to work on a family skill building activity and identify a family goal. The CDCES joined each dyad’s breakout room to discuss the goals. PWD and family member/support person participants assigned to Family-DSMES received a “Family Goal Setting Workbook” which had instructions for family skill building activities and family goal setting. Family skill building activities were designed to improve support for the PWD’s self-care goals and were adapted from an intervention proven efficacious in improving helpful family involvement among adults with T2DM [41]. The family goal specified the family member’s/support person’s commitment to engage in a specific behavior to facilitate the success of the PWD’s SMART goal. CDCESs were trained to ensure the family goal was relevant to supporting the PWD’s behavioral SMART goal. For instance, a PWD might set a SMART goal to “walk either outside or at the mall (if weather is bad) 20 min 4 days a week,” and the family member’s/support person’s goal may be “to walk with the PWD at least twice a week”; in another dyad, a PWD may set a SMART goal to “check glucose twice a day for 5 days,” and the family member’s/support person’s goal may be to “lay her glucometer next to the coffee pot each night.” The protocol publication has additional details on family goal setting [39].

### 2.4. Process data

After each DSMES session, the CDCES completed a 1-page document that included information on attendance, engagement, and start and end times. When the session included goal setting (sessions three, seven, and ten), the CDCES also documented each goal and rated fidelity per predetermined criteria (detailed below). CDCESs submitted documents to study staff within 24 h. We analyzed these data quarterly during the study to identify areas in need of CDCES retraining and at study completion to inform evaluation of implementation characteristics of Family- and Standard-DSMES. Measures examined included attendance, engagement, class time, and fidelity of goal setting. After study completion, a person not involved in the study reviewed a random selection of 20 % of goals and rated them on the same fidelity scale for an objective fidelity rating. This independent rater did not have access to the CDCES’ ratings.

### 2.5. Outcomes measures

#### 2.5.1. Primary biometric outcome

HbA1c. The primary outcome of HbA1c was collected by finger stick blood collection and analyzed using the Siemens DCA Vantage [42].

#### 2.5.2. Secondary biometric outcomes

BMI was computed using weight measured with calibrated digital scale and height measured using stadiometer. Blood pressure was measured twice with the PWD seated and then averaged. If there was a difference of 5 mmHg or greater in either diastolic or systolic blood pressure between the first and second measurement, two additional measurements were completed and all four were averaged. Non-fasting blood glucose, total cholesterol, high-density lipoprotein (HDL), low-density lipoprotein (LDL), and triglycerides were collected via finger prick and measured using an Abbott Cholestech LDX with the Cholestech-LDX Lipid Profile-Glu Cassette.

#### 2.5.3. Secondary behavioral outcomes

Frequency of diabetes self-care behaviors was assessed using pairs of items representing areas of self-care behaviors from the Summary of Diabetes Self-Care Activities (SDSCA) questionnaire; higher scores on the SDSCA measures indicate a higher frequency of the diabetes self-care behavior [43]. Adherence to glucose-lowering medication was assessed using the Adherence to Refills and Medications Scale for Diabetes (ARMS-D) [44]. Lower scores on the ARMS-D indicate better adherence to prescribed glucose-lowering medication [44]

#### 2.5.4. Secondary psychosocial outcomes

The Diabetes Management Self-Efficacy Scale (DMSES) was used to assess diabetes self-efficacy, or how confident the PWD felt in their ability to manage their diabetes; higher scores indicate greater confidence in the ability to manage diabetes [45]. Diabetes-related emotional distress was measured using the Problem Areas in Diabetes Five Item Scale (PAID-5), with lower scores indicating less diabetes-related emotional distress [46]. Diabetes-related quality of life was assessed using the DAWN2 Impact of Diabetes Profile (DIDP), with lower scores indicating better diabetes-related quality of life [47]. Diabetes-related helpful and harmful family/friend involvement in the PWD’s diabetes was assessed using the Family Involvement in Diabetes Self-Care (FIAD) measure; higher scores indicate greater helpful or harmful family/friend involvement [48]

### 2.6. Analysis

SAS 9.4 [49] was used for data cleaning and validation, producing descriptive summaries, and comparing study arms on baseline characteristics. Multiple imputation, models for comparative effectiveness, and exploration of heterogeneity of treatment effects, were completed in R [50]. The comparative effectiveness analyses were planned with sufficient power (≥80 %) to detect clinically meaningful differences (e.g., between-arm difference in HbA1c change of 0.40 % to 0.46 %) at two-sided α = 0.05. All randomized PWDs were included in analyses. A Bonferroni adjustment was applied for hypothesis tests for repeated tests within the same outcome for between-arm comparisons. The width of confidence intervals for within-arm change was also Bonferroni adjusted.

#### 2.6.1. Descriptive statistics

Demographic characteristics, covariates, and outcomes were summarized at each time of measurement. Baseline characteristics of PWDs in the study arms were compared to assess the effectiveness of the random assignment procedure. See Table 1 for between-arm differences in baseline covariates. Patterns and frequency of missing values were assessed overall and by study arm

#### 2.6.2. Process data

We examined attendance, engagement, class time, and fidelity of goal setting across study arms and across CDCESs. Our goals were to 1) determine if expected differences and similarities between the study arms were evident, 2) describe class time required for goal setting in each study arm, and 3) ensure consistency in class time and high fidelity across CDCESs. We expected family member/support person attendance in Family-DSMES but not in Standard-DSMES, but we did not expect differences in PWD attendance across arms. We expected family goal setting in Family-DSMES and not Standard-DSMES, but we did not expect differences in fidelity to SMART goal setting for PWDs across arms. We expected goal setting sessions to be longer in Family-DSMES, but we did not expect differences across arms in duration of didactic sessions without goal setting. We used summary statistics, graphical depictions, and tests of difference (e.g., t-tests) to analyze these data

Attendance was assessed by the CDCES as the presence of the enrolled PWD and, separately, the enrolled family member/support person. We examined attendance at the levels of the session and the individual. Engagement was assessed by the CDCES for each PWD as 1 = fully engaged (e.g., answering questions), 2 = moderately engaged (e.g., not speaking but camera on and attentive), and 3 = not engaged (e.g., not speaking with camera off or visibly distracted/doing other things during the session). We examined engagement at the level of the session. Fidelity for PWDs’ SMART goals was assessed by CDCESs (all goals) and an independent rater (20 %) on a scale of 0 = no goal set, 1 = not a behavioral goal, 2 = behavioral goal but not time-bound or specific, and 3 = behavioral SMART goal. Family-DSMES family goals were assessed by both CDCES (all goals) and an independent rater (20 %) on a scale of 0 = no family goal, 1 = reminding PWD to do the goal or vague (e.g., “help” with goal), 2 = specific behavior identified/regular check in scheduled, and 3 = doing goal together. We examined fidelity of goal setting at the level of the session. Class time was calculated in minutes for each session using start and end times reported by the CDCES. We also calculated time spent after the session with individual PWD or family member/support person participants (“chat time”) based on CDCES report. We examined class time several ways: comparing didactic sessions with goal setting sessions, comparing across CDCESs, comparing across arms, and total time spent in DSMES for each PWD.

#### 2.6.3. Outcomes analysis

Potential clustering effects due to PWDs nested within DSMES groups or clinics were explored using mixed effects models. Between-cluster variances were very small and resulted in estimation errors. Therefore, generalized estimating equations (GEE) models were used to assess changes in all measures. The models included time, study arm, two-way interaction terms (time × study arm), and the covariates: race/ethnicity, age, gender, and insulin use at baseline. For HbA1c, BMI, blood pressure, and diabetes distress, GEE models were specified with an identity link function, assuming a Gaussian distribution for the response variable and an unstructured working correlation structure. For the binary measure related to smoking over the past week, the GEE model used a logit link function, assuming a binomial distribution for the response variable, and an exchangeable correlation structure, assuming a constant correlation among repeated observations

The GEE models were used for parameter estimates, standard errors, test statistics, and p-values. Adjusted least-squares means were estimated for each measure for time-specific point estimates, within-arm changes, and between-arm differences in change. Pooled pairwise comparisons were adjusted for multiplicity using the Bonferroni correction, and 95 % confidence intervals were derived using appropriate standard error calculations.

#### 2.6.4. Heterogeneity analyses for HbA1c and diabetes-related emotional distress

We examined potential heterogeneity of treatment effects (HTE) across: Clinic location (rural/urban), race/ethnicity (non-Hispanic White, non-Hispanic African American or Black), age (18–64/≥65 years old), gender (male/female), PWD-family member/support person relationship (romantic partner/other relationship), and PWD-family member/support person living status (living separately/co-residing). For each subgroup, change from baseline to immediate post-intervention and its 95 % confidence interval was estimated using the previously described GEE analysis of the entire sample with additional three-way and two-way interaction terms needed for the HTE estimates

#### 2.6.5. Missing data

At the immediate post-intervention, the missingness of measures ranged from 9.8 % to 26.5 %. The lowest missingness was seen in systolic and diastolic blood pressure and smoking over the past week. The highest missingness was for LDL values. Table 1 shows the number of complete observations for each variable on each occasion. To address missing data, we used the random forest method within multiple imputation by chained equations (MICE) [51]. A total of 20 imputed datasets were generated, consistent with fraction of missing information (FMI) based guidance that recommends setting the number of imputations to at least 100 × FMI to ensure reproducibility [52]. Model estimates were pooled across these imputed datasets using Rubin’s Rules [53,54].

## 3. Results

### 3.1. Recruitment

**Fig. 1.**
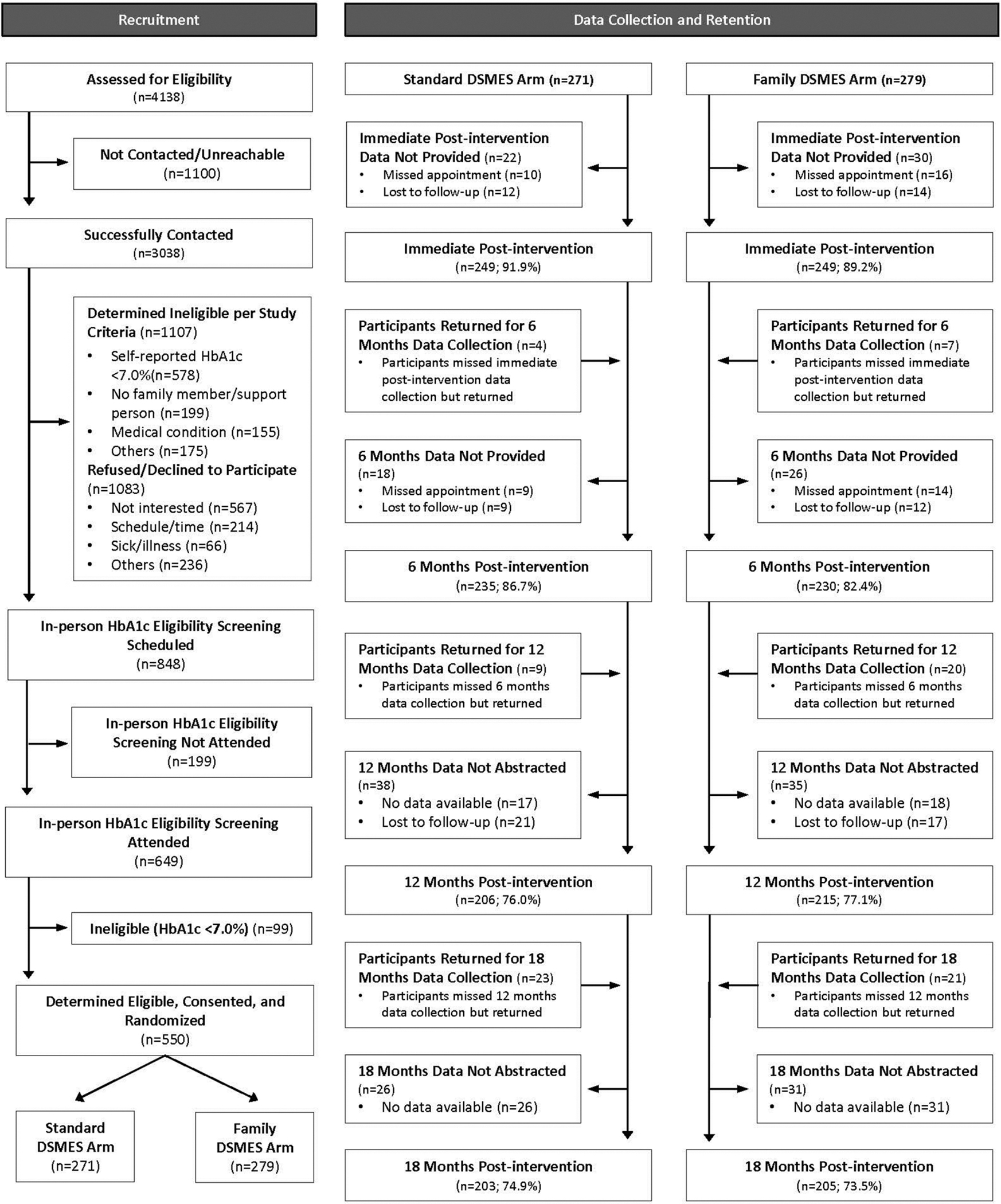
Study recruitment, enrollment, randomization, and retention flow diagram.

550 PWDs were enrolled and randomized to the Standard-DSMES arm (271 PWDs) or the Family-DSMES arm (279 PWDs). Fig. 1 provides additional information on recruitment and the total number of PWDs from each arm who provided data at each time point.

### 3.2. PWD characteristics

Table 1 reports the characteristic of the sample by study arm and overall. The sample was majority non-Hispanic African American or Black (54 %), between the ages of 18 and 64 (77.1 %), and female (70.1 %). Over half (53.2 %) were currently treated with insulin; mean HbA1c was 9.2 %, and mean BMI was 37.1 at baseline.

### 3.3. Process data

Attendance for PWDs was median 70 % interquartile range (IQR) [20, 90 %], with mean attendance slightly higher in Family-DSMES than Standard-DSMES (62 % vs. 57 %, *p* = 0.096). Seventy percent (70 %) of PWDs completed at least five sessions in the Family-DSMES arm versus 64 % in the Standard-DSMES arm. As expected, family members/support persons were much more likely to attend Family-DSMES than Standard-DSMES sessions (mean 55 % vs. 3 %, *p* < 0.001). Ninety-four percent (94 %) of PWDs in the Family-DSMES arm attended at least one session with their family member/support person. Engagement for PWDs per session was similar across arms (*p* = 0.319) with 85 % fully engaged, 13 % moderately engaged, and 2 % not engaged. In the Family-DSMES arm, family members’/support persons’ per session engagement was 75 % fully engaged, 19 % moderately engaged, and 5 % not engaged. Fidelity for PWDs’ goal setting was high as assessed by CDCESs (98 % were behavioral SMART goals) and the independent rater (95 %). Similarly, in Family-DSMES, family goals were assessed as either “specific behavior identified/regular check in scheduled” or “doing goal together” 86 % of the time by CDCESs and 87 % by the independent rater. Class time was on average 54 min (SD = 6.2 min, IQR [49, 57 min]) with no difference between study arms for didactic classes (*p* = 0.145). Family-DSMES goal setting classes lasted a mean 9 min longer than Standard-DSMES goal setting classes (95 % CI: 6.1, 12.1, *p* < 0.001). Post-class “chat time” was on average 5 min and consistent across arms (*p* = 0.233) and didactic versus goal setting class types (*p* = 0.228). There was consistency in class lengths across CDCESs, with each CDCESs’ mean class time ranging 50 to 57 min. At the level of the PWD, total class time provided was intended to be 600 min (ten hours) or less; observed class time was mean 544 min IQR [426, 563] for Family-DSMES and 504 min IQR [485, 521] for Standard-DSMES (*p* = 0.002) with an average of 28 more minutes total spent goal setting for Family-DSMES as compared to Standard-DSMES (*p* < 0.001) and no between-arm difference in total didactic class time (*p* = 0.145).

### 3.4. Effects of standard and family DSMES on outcomes

Table 2 shows estimated change from baseline to each post-intervention time point for all outcomes by study arm; p-values are for tests of whether the change from baseline was statistically significant.

#### 3.4.1. HbA1c

Both the Family-DSMES and Standard-DSMES arms showed significant reductions in HbA1c at all post-intervention time points compared to baseline. The Standard-DSMES arm PWD experienced HbA1c decreases of 0.77 % (95 % CI: − 1.04, − 0.51, *p* < 0.001), 0.73 % (95 % CI: − 1.09, − 0.37, *p* < 0.001), and 0.83 % (95 % CI: − 1.21, − 0.44, *p* < 0.001) at immediate, 6, and 12 months post-intervention, respectively, with all changes statistically significant. The Family-DSMES arm showed HbA1c reductions of 0.69 % (95 % CI: − 0.99, − 0.40, *p* < 0.001), 0.45 % (95 % CI: − 0.79, − 0.10, *p* = 0.004), and 0.52 % (95 % CI: − 0.90, − 0.15, *p* = 0.002) at these respective time points, with all changes statistically significant. Both interventions were effective in reducing HbA1c levels

#### 3.4.2. Other biometric outcomes

The Standard-DSMES arm showed a statistically significant reduction in non-fasting blood glucose of –22.73 (95 % CI: − 36.42, − 9.03, *p* < 0.001) at immediate post-intervention compared to baseline (Table 2). There were not statistically significant differences for any of the other biometric outcomes for either the Standard-DSMES or Family-DSMES PWDs

#### 3.4.3. Behavioral outcomes

Both the Standard-DSMES and Family-DSMES arms showed significant improvements in diabetes self-care behaviors at immediate post-intervention compared to baseline, apart from smoking over the past week (Table 2). Both the Standard-DSMES (− 0.89, 95 % CI: − 1.56, − 0.23, *p* = 0.005) and the Family-DSMES (− 1.33, 95 % CI: − 2.12, − 0.53, *p* < 0.001) PWDs saw significant improvements in glucose-lowering medication adherence at immediate post-intervention compared to baseline (Table 2)

#### 3.4.4. Psychosocial outcomes

Both the Standard-DSMES and Family-DSMES arms showed significant (*p* < 0.001) improvements in diabetes self-efficacy at immediate post-intervention compared to baseline. Family-DSMES PWDs had a slightly greater increase in diabetes self-efficacy of 0.74 points (95 % CI: 0.42, 1.05) compared to the Standard-DSMES PWDs with an increase of 0.53 points (95 % CI: 0.23, 0.84)

PWDs in the Family-DSMES arm (0.39, 95 % CI: 0.22, 0.56, *p* < 0.001) experienced an increase in diabetes-related helpful family involvement. PWDs in the Family-DSME arm (− 0.13, 95 % CI: − 0.23, − 0.03, *p* = 0.007) experienced a decrease in diabetes-related harmful family involvement (Table 2).

Both the Standard-DSMES and Family-DSMES arms showed significant reductions in diabetes-related emotional distress at immediate post-intervention compared to baseline. The Standard-DMSES arm PWDs experienced a reduction of 1.03 points (95 % CI: − 1.86, − 0.20, *p* = 0.011), while the Family-DSMES arm demonstrated a reduction of 1.27 points (95 % CI: − 2.06, − 0.47, *p* < 0.001).

PWDs in the Standard-DSMES arm (− 0.33, 95 % CI: − 0.55, − 0.11, *p* = 0.002) saw an improvement in diabetes-related quality of life from baseline to the immediate post-intervention time point (Table 2). No significant difference was found among the Family-DSMES PWDs.

### 3.5. Comparative effectiveness across standard and family-DSMES on outcomes

There were not statistically significant between-arm differences found between the Family-DSMES and Standard-DSMES arms for the biometric outcomes or behavioral outcomes. For diabetes-related helpful family involvement (FIAD), the improvement in the Family-DSMES arm was significantly greater than the improvement in the Standard-DSMES arm (0.24, 95 % CI: 0.03, 0.45, *p* = 0.024). None of the other psychosocial outcomes had a statistically significant between-arm difference.

### 3.6. Heterogeneous treatment effects on HbA1c and diabetes-related emotional distress

**Fig. 2.**
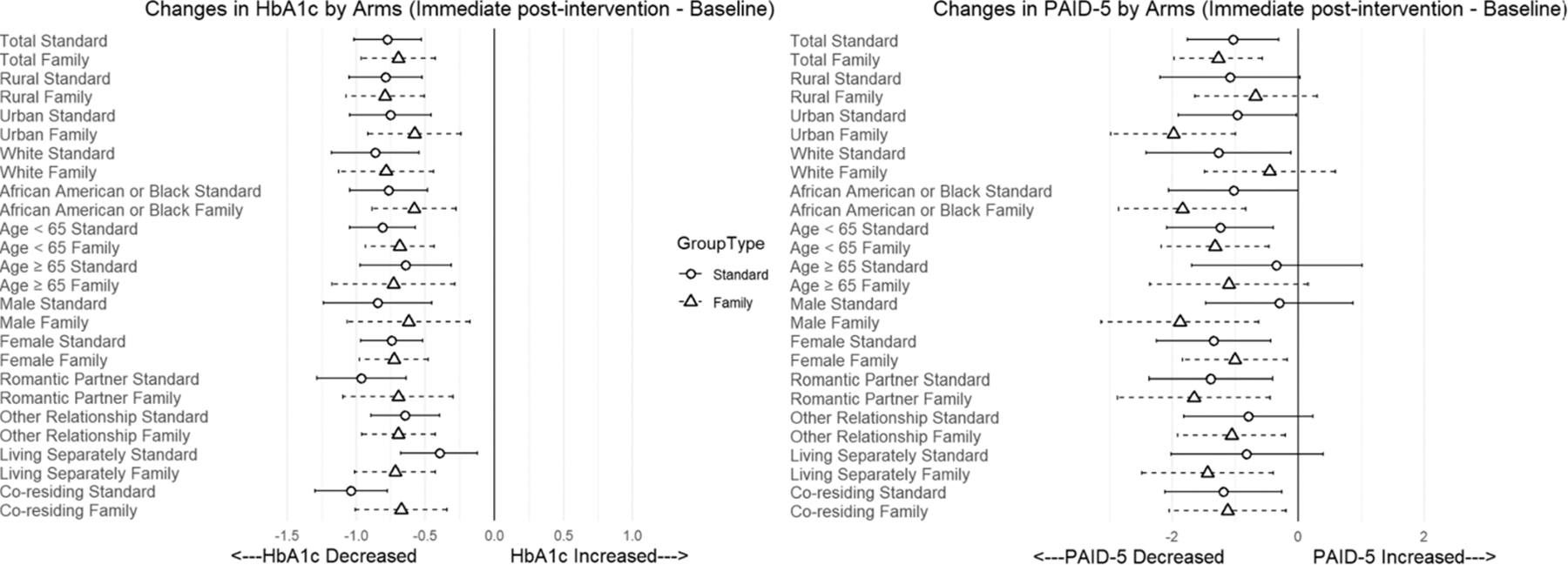
Forest plots for heterogeneity analyses.

We explored treatment heterogeneity across various PWD subgroups; however, it is of note that the study was not powered to provide confirmatory testing of HTEs. Forest plots (Fig. 2) summarize the subgroup effects and illustrate the variation in change in HbA1c and diabetes-related emotional distress across different demographic and relationship contexts. Although there is clear variation in change across groups, only the model for HbA1c and PWD- family member/support person living status showed a significant time-by-study arm-by-sub-group interaction (*p* = 0.024). The results for HbA1c underscore that both Standard-DSMES and Family-DSMES can lead to significant improvements in HbA1c; however, there is some variability by subgroup as seen in Fig. 2. Overall, both the Standard-FDSMES and Family-FDSMES showed improvement in diabetes-related emotional distress (PAID-5; Fig. 2); however, some subgroups seem to have greater reductions in diabetes-related emotional distress when participating in the Family-DSMES intervention, although there were not significant differences. Particularly, the Family-DSMES arm showed greater improvements in diabetes-related emotional distress for PWDs from urban areas, African American or Black PWDs, male PWDs, and PWDs who lived separately from their family member/support person participant.

## 4. Discussion

PWDs in both the Family-DSMES and Standard-DSMES achieved statistically and clinically significant reductions in HbA1c at all post-intervention time points compared to baseline. However, there were no significant between-arm differences. The lack of between-arm difference in HbA1c change and most other outcomes was not expected and is inconsistent with prior literature in single populations with culturally adapted curriculum [28,55,56]. PWDs in the Family-DSMES arm reported significant increases in diabetes-related helpful family involvement, and their improvements were significantly greater than in the Standard-DSMES arm. This improvement in diabetes-related helpful family involvement is important because prior studies have demonstrated improvements in helpful family involvement to have a long-term influence on sustained clinical outcomes [57,58].

The PWDs in the study were diverse, with 63 % being from minoritized populations. Additionally, the study was conducted in both rural and urban clinics with 55 % of the PWDs being in rural clinics. The Family-DSMES and Standard-DSMES models both showed improvement in HbA1c for the subgroups in the heterogeneity models, illustrating that both models have a positive effect on HbA1c. However, for diabetes-related emotional distress, PWDs in the Family-DSMES arm who were urban, who were African American or Black, who were male, or who lived separately from their family member/support person participant showed some evidence of greater improvement. These results illustrate that for some subgroups, there is a potential psychological benefit in the form of a reduction in diabetes-related emotional distress from Family-DSMES models. Reductions in diabetes-related emotional distress have been linked to improvements in future glycemic outcomes and medication adherence, and reductions in the risk of mortality, poor disease management, and diabetes-related complications [59,60]. Thus, providing DSMES using a family model may improve short- and long-term outcomes for these subgroups of PWDs.

This study does have some limitations. The study was implemented through telehealth, and results may have differed if both models had been offered via in-person group sessions. Our evaluation of Family-DSMES was potentially limited by variability in family member participation. Although our sample is diverse in race/ethnicity and rurality, it may not be generalizable to all clinical populations of PWDs. Additionally, some participants did need assistance with cellular devices and internet services, which may limit the scaleability and sustainability of the intervention, particularly in resource-limited environments. Repeated survey measures were limited to immediate post-intervention data collection, which did not allow us to evaluate sustained benefits of Family-DSMES on behavioral and psychosocial outcomes. The curriculum was not culturally adapted which may potentially limit the effectiveness of Family-DSMES for populations with specific dietary, familial, or social traditions. It is possible that our approach to missing data may also pose a limitation. We addressed missing data using multiple imputation with MICE and a random forest approach. Data were imputed in wide format, with repeated measures of the same variable represented as separate columns. For the GEE analysis, each imputed data set was transformed to long form with repeated measures represented as separate rows. Although this was a pragmatic approach given wide availability of this imputation approach across many software platforms, it may be problematic that the imputation model used the data in wide form whereas the analysis model used the data in long form emphasizing the within-person nature of the repeated measures. It should also be noted that several between-arm comparisons for the secondary outcomes are reported and although Bonferroni adjustments were applied within outcomes, no other adjustments were made for multiple comparisons. Despite these limitations, the study provides important findings related to the ability to offer Family-DSMES in a rural and diverse population through telehealth with high fidelity, filling several important gaps in the literature. Our methods for delivery of DSMES via telehealth, and therefore our results, are highly generalizable and can inform clinical practice and future research.

This is the first fully powered RCT to compare Family-DSMES to Standard-DSMES in diverse populations using telehealth. The results of the study demonstrate the ability to deliver Family-DSMES via telehealth with high fidelity, high attendance, and engagement for both PWD and family members/support persons and that Family-DSMES is achievable within the ten hours covered by insurance while meeting established national standards. CDCESs were able to facilitate goal setting for both the PWDs and family members/support persons using breakout rooms without increasing time requirements over Standard-DSMES. Despite prior literature expressing concern over the limitations of the digital divide [61,62], the results of this study demonstrate the ability to engage rural and diverse PWDs in a telehealth intervention with high fidelity to achieve clinically and statistically significant improvements in HbA1c and diabetes-related emotional distress.

## Figures and Tables

**Table 1:** Descriptive statistics.

	Standard (n = 271) Col. % (Freq.) or *Mean (SD)*	Family (n = 279) Col. % (Freq.) or *Mean (SD)*	Total (n = 550) Col. % (Freq.) or *Mean (SD)*
**Sociodemographics**			
**Race**			
Non-Hispanic White	37.64 (102)	37.28 (104)	37.45 (206)
Non-Hispanic African American or Black	54.24 (147)	53.76 (150)	54.00 (297)
Hispanic and other	8.12 (22)	8.96 (25)	8.55 (47)
**Age**			
18–64	77.12 (209)	77.06 (215)	77.09 (424)
>65	22.88 (62)	22.94 (64)	22.91 (126)
**Gender**			
Male	29.63 (80)	30.22 (84)	29.93 (164)
Female	70.37 (190)	69.78 (194)	70.07 (384)
**Insulin Use**			
Don’t use insulin	49.62 (131)	44.16 (121)	46.84 (252)
Use insulin	50.38 (133)	55.84 (153)	53.16 (286)
**Location**			
Rural	55.35 (150)	54.84 (153)	55.09 (303)
Urban	44.65 (121)	45.16 (126)	44.91 (247)
**Type of Relationship**			
Romantic partner	40.15 (108)	35.38 (98)	37.73 (206)
Other type of relationship	59.85 (161)	64.62 (179)	62.27 (340)
**Cohabitating Status**			
Living separately	41.67 (110)	46.13 (125)	43.93 (235)
Co-residing	58.33 (154)	53.87 (146)	56.07 (300)
**Biometrics**			
**Hemoglobin A1c (%)**			
Baseline (n = 550)	*9.3 (1.9)*	*9.2 (1.8)*	*9.2 (1.9)*
Immediate post-intervention (n = 495)	*8.5 (1.9)*	*8.5 (1.9)*	*8.5 (1.9)*
6 months post-intervention (n = 464)	*8.5 (2.1)*	*8.7 (2.0)*	*8.6 (2.1)*
12 months post-intervention (n = 421)	*8.4 (2.1)*	*8.6 (2.1)*	*8.5 (2.1)*
**BMI**			
Baseline (n = 546)	*36.69 (8.52)*	*37.57 (9.58)*	*37.14 (9.08)*
Immediate post-intervention (n = 485)	*36.48 (8.63)*	*37.59 (9.50)*	*37.03 (9.08)*
**Systolic Blood Pressure**			
Baseline (n = 548)	*133.24 (19.68)*	*134.29 (22.88)*	*133.77 (21.34)*
Immediate post-intervention (n = 496)	*132.63 (18.21)*	*135.79 (21.05)*	*134.21 (19.72)*
6 months post-intervention (n = 462)	*133.48 (18.96)*	*134.91 (22.33)*	*134.19 (20.67)*
**Diastolic Blood Pressure**			
Baseline (n = 548)	*81.10 (11.98)*	*81.32 (13.07)*	*81.21 (12.53)*
Immediate post-intervention (n = 496)	*80.36 (11.79)*	*82.08 (13.55)*	*81.22 (12.72)*
6 months post-intervention (n = 462)	*80.45 (11.92)*	*80.68 (14.62)*	*80.56 (13.30)*
**Glucose**			
Baseline (n = 543)	*216.14 (89.22)*	*212.00 (87.95)*	*214.04 (88.52)*
Immediate post-intervention (n = 478)	*193.36 (79.51)*	*203.58 (88.24)*	*198.41 (84.00)*
**Total Cholesterol**			
Baseline (n = 545)	*164.47 (46.46)*	*166.99 (51.58)*	*165.74 (49.09)*
Immediate post-intervention (n = 484)	*163.57 (47.36)*	*164.00 (47.86)*	*163.78 (47.56)*
**HDL**			
Baseline (n = 536)	*38.58 (14.52)*	*38.68 (13.63)*	*38.63 (14.06)*
Immediate post-intervention (n = 477)	*39.88 (14.72)*	*38.53 (13.40)*	*39.21 (14.09)*
**LDL**			
Baseline (n = 459)	*94.26 (36.99)*	*93.67 (44.69)*	*93.96 (41.05)*
Immediate post-intervention (n = 404)	*91.35 (37.84)*	*90.27 (36.61)*	*90.82 (37.20)*
**Triglycerides**			
Baseline (n = 545)	*192.86 (116.85)*	*204.32 (122.50)*	*198.66 (119.77)*
Immediate post-intervention (n = 483)	*181.49 (99.09)*	*201.43 (122.07)*	*191.44 (111.49)*
**Survey Measures**			
**Diabetes Self-Care Behaviors (SDSCA): General Diet**			
Baseline (n = 524)	*2.95 (2.20)*	*3.10 (2.33)*	*3.03 (2.26)*
Immediate post-intervention (n = 470)	*4.40 (1.89)*	*4.53 (2.02)*	*4.46 (1.96)*
**Diabetes Self-Care Behaviors (SDSCA): Fruits and Vegetables**			
Baseline (n = 534)	*3.22 (2.24)*	*3.47 (2.45)*	*3.34 (2.35)*
Immediate post-intervention (n = 480)	*4.14 (2.14)*	*4.21 (2.13)*	*4.18 (2.13)*
**Diabetes Self-Care Behaviors (SDSCA): Fatty Foods**			
Baseline (n = 535)	*3.49 (2.04)*	*3.68 (2.11)*	*3.59 (2.08)*
Immediate post-intervention (n = 481)	*3.14 (1.86)*	*3.11 (2.02)*	*3.12 (1.94)*
**Diabetes Self-Care Behaviors (SDSCA): Exercise**			
Baseline (n = 538)	*1.90 (2.06)*	*2.05 (1.99)*	*1.98 (2.02)*
Immediate post-intervention (n = 482)	*2.72 (2.11)*	*3.28 (2.26)*	*3.00 (2.20)*
**Diabetes Self-Care Behaviors (SDSCA): Blood Glucose Testing**			
Baseline (n = 529)	*3.62 (2.71)*	*4.08 (2.79)*	*3.85 (2.76)*
Immediate post-intervention (n = 469)	*4.87 (2.34)*	*5.15 (2.08)*	*5.01 (2.21)*
**Diabetes Self-Care Behaviors (SDSCA): Foot Care**			
Baseline (n = 540)	*3.55 (2.62)*	*3.51 (2.56)*	*3.53 (2.59)*
Immediate post-intervention (n = 477)	*4.53 (2.42)*	*4.55 (2.51)*	*4.54 (2.46)*
**Diabetes Self-Care Behaviors (SDSCA): Smoking over the Past Week**			
Baseline (n = 550)	*0.16 (0.37)*	*0.21 (0.41)*	*0.19 (0.39)*
Immediate post-intervention (n = 496)	*0.14 (0.35)*	*0.22 (0.41)*	*0.18 (0.38)*
**Glucose-Lowering Medication Adherence (ARMS-D)**			
Baseline (n =524)	*17.17 (4.77)*	*17.43 (5.75)*	*17.30 (5.29)*
Immediate post-intervention (n =471)	*16.37 (4.44)*	*16.07 (4.18)*	*16.22 (4.31)*
**Diabetes Self-Efficacy (DMSES)**			
Baseline (n = 521)	*6.90 (1.94)*	*6.80 (2.03)*	*6.85 (1.98)*
Immediate post-intervention (n = 458)	*7.40 (1.87)*	*7.55 (1.90)*	*7.48 (1.88)*
**Diabetes-Related Helpful Family Involvement (FIAD)**			
Baseline (n =542)	*2.15 (1.00)*	*2.21 (1.07)*	*2.18 (1.04)*
Immediate post-intervention (n =485)	*2.27 (1.05)*	*2.62 (1.10)*	*2.45 (1.09)*
**Diabetes-Related Harmful Family Involvement (FIAD)**			
Baseline (n =541)	*1.76 (0.73)*	*1.72 (0.66)*	*1.74 (0.70)*
Immediate post-intervention (n =482)	*1.66 (0.67)*	*1.59 (0.62)*	*1.62 (0.64)*
**Diabetes-Related Emotional Distress (PAID-5)**			
Baseline (n =539)	*7.08 (6.02)*	*7.56 (5.83)*	*7.32 (5.92)*
Immediate post-intervention (n =478)	*6.14 (5.55)*	*6.29 (5.88)*	*6.21 (5.71)*
**Diabetes-Related Quality of Life (DIDP)**			
Baseline (n =537)	*4.60 (1.09)*	*4.42 (1.15)*	*4.51 (1.13)*
Immediate post-intervention (n =477)	*4.26 (1.20)*	*4.28 (1.17)*	*4.27 (1.18)*

Note: SD=standard deviation.

**Table 2:** Changes from baseline to each post-intervention occasion.

	Standard Arm		Family Arm	
	Change	Adjusted CI	Change	Adjusted CI
**Hemoglobin A1c**				
*Immediate post-intervention – Baseline*	−0.77	[−1.04, −0.51]	−0.69	[−0.99, −0.40]
*6 months post-intervention – Baseline*	−0.73	[−1.09, −0.37]	−0.45	[−0.79, −0.10]
*12 months post-intervention – Baseline*	−0.83	[−1.21, −0.44]	−0.52	[−0.90, −0.15]
**BMI**				
*Immediate post-intervention – Baseline*	−0.18	[−0.88, 0.53]	−0.18	[−0.95, 0.60]
*6 months post-intervention – Baseline*	−0.42	[−1.19, 0.34]	−0.34	[−1.28, 0.61]
**Systolic Blood Pressure**				
*Immediate post-intervention – Baseline*	−0.59	[−3.56, 2.39]	1.51	[−1.91, 4.92]
*6 months post-intervention – Baseline*	0.24	[−3.06, 3.54]	0.35	[−3.50, 4.20]
**Diastolic Blood Pressure**				
*Immediate post-intervention – Baseline*	−0.75	[−2.66, 1.17]	0.63	[−1.32, 2.58]
*6 months post-intervention – Baseline*	−0.71	[−2.89, 1.47]	−0.74	[−2.95, 1.47]
**Glucose**				
*Immediate post-intervention – Baseline*	−22.73	[−36.42, −9.03]	−8.49	[−22.91, 5.93]
**Total Cholesterol**				
*Immediate post-intervention – Baseline*	−1.04	[−7.59, 5.52]	−3.00	[−8.93, 2.94]
**HDL**				
*Immediate post-intervention – Baseline*	1.20	[−0.43, 2.83]	−0.21	[−1.90, 1.48]
**LDL**				
*Immediate post-intervention – Baseline*	−2.20	[−8.56, 4.16]	−3.03	[−9.16, 3.10]
**Triglycerides**				
*Immediate post-intervention – Baseline*	−10.61	[−27.23, 6.00]	−2.44	[−18.86, 13.97]
**Diabetes Self-Care Behaviors (SDSCA): General Diet**				
*Immediate post-intervention – Baseline*	1.46	[1.14, 1.78]	1.40	[1.07, 1.74]
**Diabetes Self-Care Behaviors (SDSCA): Fruits and Vegetables**				
*Immediate post-intervention – Baseline*	0.92	[0.57, 1.28]	0.74	[0.38, 1.11]
**Diabetes Self-Care Behaviors (SDSCA): Fatty Foods**				
*Immediate post-intervention – Baseline*	−0.36	[−0.69, −0.04]	−0.56	[−0.90, −0.23]
**Diabetes Self-Care Behaviors (SDSCA): Exercise**				
*Immediate post-intervention – Baseline*	0.83	[0.52, 1.15]	1.16	[0.84, 1.49]
**Diabetes Self-Care Behaviors (SDSCA): Blood Glucose Testing**				
*Immediate post-intervention – Baseline*	1.27	[0.89, 1.65]	1.07	[0.72, 1.41]
**Diabetes Self-Care Behaviors (SDSCA): Foot Care**				
*Immediate post-intervention – Baseline*	1.03	[0.66, 1.40]	1.10	[0.73, 1.47]
**Diabetes Self-Care Behaviors (SDSCA): Smoking over the Past Week^a^**				
*Immediate post-intervention – Baseline*	0.79	[0.60, 1.05]	0.95	[0.75, 1.20]
**Glucose-Lowering Medication Adherence (ARMS-D)**				
*Immediate post-intervention – Baseline*	−0.89	[−1.56, −0.23]	−1.33	[−2.12, −0.53]
**Diabetes Self-Efficacy (DMSES)**				
*Immediate post-intervention – Baseline*	0.53	[0.23, 0.84]	0.74	[0.42, 1.05]
**Diabetes-Related Helpful Family Involvement (FIAD)** ^b^				
*Immediate post-intervention – Baseline*	0.15	[−0.01, 0.31]	0.39	[0.22, 0.56]
**Diabetes-Related Harmful Family Involvement (FIAD)**				
*Immediate post-intervention – Baseline*	−0.10	[−0.22, 0.01]	−0.13	[−0.23, −0.03]
**Diabetes-Related Emotional Distress (PAID-5)**				
*Immediate post-intervention – Baseline*	−1.03	[−1.86, −0.20]	−1.27	[−2.06, −0.47]
**Diabetes-Related Quality of Life (DIDP)**				
*Immediate post-intervention – Baseline*	−0.33	[−0.55, −0.11]	−0.14	[−0.33, 0.05]

Note: Models adjusted for time, study arm, time*study arm, race/ethnicity, age, gender, and insulin use. CI=confidence interval.

a : The estimated changes of outcome “Diabetes Self-Care Behaviors (SDSCA): Smoking over the Past Week” are odds ratios from logistic GEE models.

b : Significant between-arm differences in change (p-value = 0.024).
